# A Retrospective Comparison of Conventional versus Transverse Mini-Incision Technique for Carpal Tunnel Release

**DOI:** 10.1155/2013/721830

**Published:** 2013-12-12

**Authors:** İsmail Gülşen, Hakan Ak, Gökhan Evcılı, Özlem Balbaloglu, Enver Sösüncü

**Affiliations:** ^1^Department of Neurosurgery, School of Medicine, Yıl University, Van, Turkey; ^2^Department of Neurosurgery, School of Medicine, Bozok University, Yozgat, Turkey; ^3^Derince Teaching and Searching Hospital, Kocaeli, Turkey; ^4^Department of Physical Therapy and Rehabilitation, School of Medicine, Bozok University, Yozgat, Turkey

## Abstract

*Background*. In this retrospective study, we aimed to compare the results of two surgical techniques, conventional and transverse mini-incision. *Materials and Methods*. 95 patients were operated between 2011 and 2012 in Bitlis State Hospital. 50 patients were operated with conventional technique and 45 of them were operated with minimal transverse incision. Postoperative complications, incision site problems, and the time of starting to use their hands in daily activities were noted. *Results*. 95 patients were included in the study. The mean age was 48. 87 of them were female and 8 were male. There was no problem of incision site in both of the two surgical techniques. Only in one patient, anesthesia developed in minimal incision technique. The time of starting to use their hands in daily activities was 22,2 days and 17 days in conventional and minimal incision technique, respectively. *Conclusion*. Two surgical techniques did not show superiority to each other in terms of postoperative complications and incision site problems except the time of starting to use their hands in daily activities.

## 1. Introduction

Carpal tunnel syndrome (CTS) is the most common entrapment neuropathy caused by the compression of the median nerve in the carpal tunnel. Its prevalence is about 0.6–3.4% in general population. It is more common in females [1]. Common complaints are painful paresthesias or burning pain in the lateral half of the hand and the radial three fingers [2, 3].

Diagnosis is performed with the history, physical and neurological examination, electromyography, and nerve conduction velocity measurements [3]. In the management of CTS, conservative procedures and surgical approaches are well defined in the literature. Surgery is kept for severe cases and in whom conservative management fails. The aim of the surgery is to release the transverse carpal ligament and thereby to increase canal volume and reduce the pressure over the median nerve [4]. Different surgical approaches are defined. These may be divided into two main categories, endoscopic and nonendoscopic procedures. Nonendoscopic procedure includes a standard open technique (conventional), a wrist-incision technique, and midpalmar-incision technique [5].

In the present study, we aimed to compare the surgical results of two nonendoscopic different techniques, in the eastern part of Turkey, retrospectively.

## 2. Materials and Methods 

This study included 200 patients who were diagnosed with CTS in Bitlis State Hospital between May 2011 and May 2012. 95 patients who did not benefit from conservative management and electromyography findings revealed moderate, moderate-severe, and severe were operated. Folders of these 95 patients were retrospectively searched for incision type, need of reoperation, scar-related problems, any intraoperative complication, and the time of starting to use their hands in daily activities. Operations were performed by two different neurosurgeons (IG, HA) according to their own experience.

### 2.1. Surgical Incision

**Figure 1:**
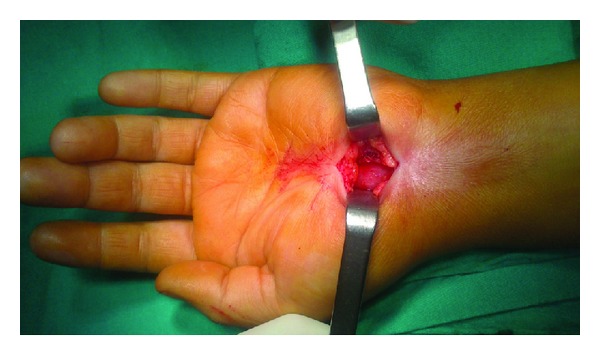
Incision line for conventional technique.

**Figure 2:**
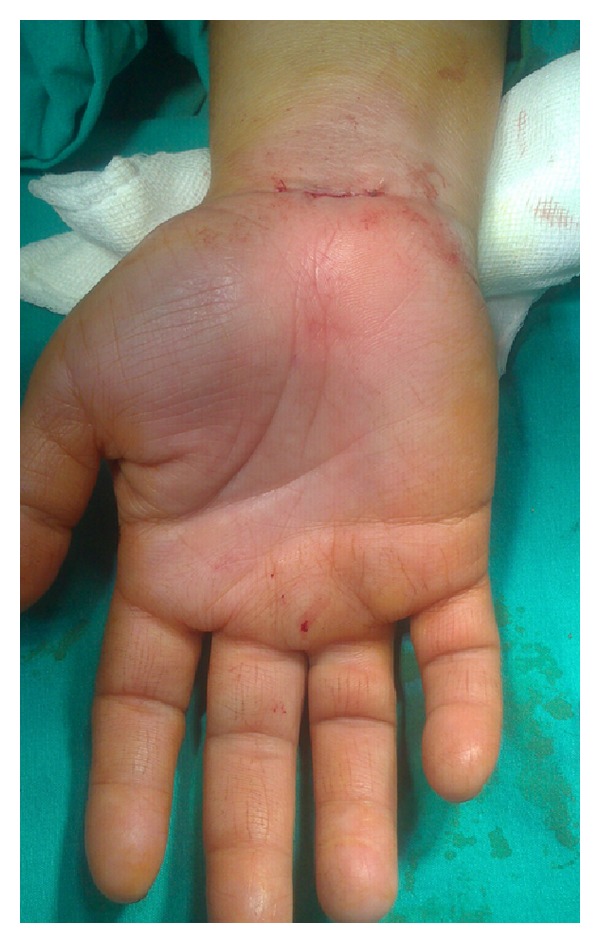
Incision line for transverse mini-incision technique.

**Figure 3:**
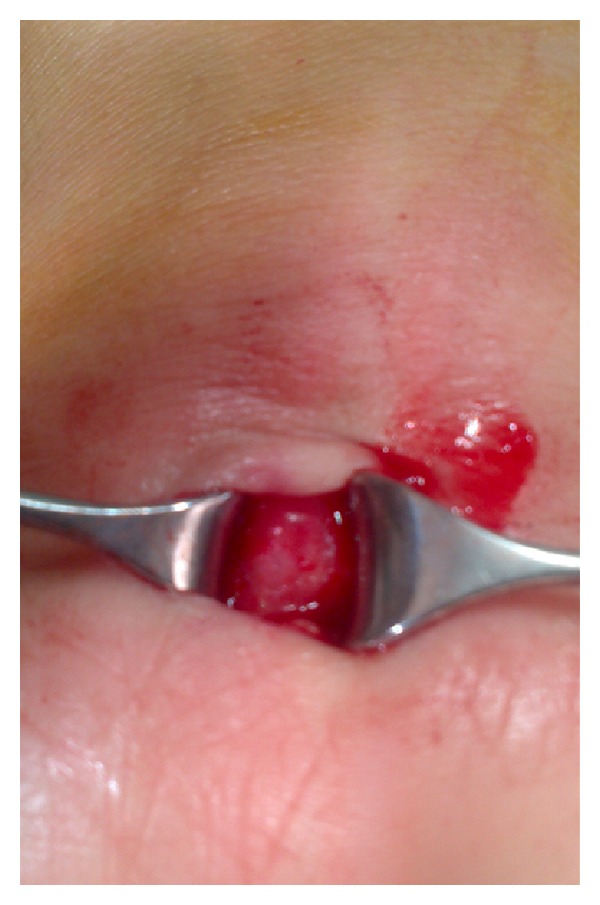
Intraoperative appearance of median nerve.

Both types of incision were made under local anesthesia. First incision type (group I, operator HA) was shortened conventional type in which an incision beginning from distal wrist crease and extending distally about 2 to 2,5 cm over the carpal canal was made (Figure 1). After that with blunt dissection transverse carpal ligament is found and cut until the distal end. Second incision type (group II, operator IG) was transverse mini-incision in which a transverse incision was made 1,5–2 cm in length about 1 cm above distal wrist crease (Figures 2 and 3). After incision, palmaris longus tendon was identified laterally to the median nerve on the anterior surface of the wrist. After that the median nerve was released blindly.

### 2.2. Results

This retrospective study included 95 patients. 87 (91,5%) of them were female and 8 (8,5%) were male. 50 of them were been operated with shortened conventional incision, group I, and 45 of them were been operated by horizontal incision, group II. The mean age was 53,2 (42–76). Mean age was 53,76 ± 9,0 years in group I and 52,60 ± 8,0 years in group II. There was no statistical difference between the two groups (*P* = 0,513).

We detected that 4 (8%) patients in the first group were rereferred with the scar tissue problem at the wound site and were treated with the topical application of mupirocin containing cream. In the second group, no problems were determined associated with the formation of scar tissue. When two groups were compared in terms of the formation of scar tissue at the wound site, there was no statistical difference (*P* = 0,07).

We determined that in a patient in the second group transverse mini-incision technique had been converted to classical conventional technique due to uncontrolled digital artery bleeding. Also, we detected ulnar artery injury in a patient in the second group. We noted that this patient complained of sensory loss in the same hand up to anesthesia about one month after operation. This patient was reoperated due to anesthesia and severe CTS findings in EMG with the classical conventional technique. Additionally, we determined that a patient in the same group reoperated due to recurrence of preoperative complaints and severe CTS findings in EMF 3 months after operation with classical conventional method. As a result, the need of surgery with classical conventional technique was determined in total of three patients in the second group. However, no statistically significant difference was observed in terms of need of classical conventional technique (*P* = 0,5). Also, there was no significant difference in terms of intraoperative complication rates (*P* = 0,47).

The use of patients' hands after operation in daily activities was 22.28 ± 9.4 days in group I and 15,96 ± 7,4 days in group II. Statistically significant difference was observed between two groups in terms of starting to use hands in daily activities (*P* = 0,02).

### 2.3. Statistical Analysis

The statistical analyses were performed using software (SPSS 18.0). Parametric values were given as a mean ± standard deviation and nonparametric values were given as a percentage. To compare parametric continuous variables, Student's *t*-test was used; to compare nonparametric continuous variables, the Mann-Whitney *U* test was used. Categorical data were compared by chi-square distribution. Two-tailed *P* values of less than 0.05 were considered to indicate statistical significance.

## 3. Discussion

Since the first description of operative treatment of CTS, which is the most common peripheral entrapment neuropathy, it has become a profession which neurology clinicians, neurosurgeons, orthopedic surgeons, and plastic surgeons deal with it [5]. According to this fact, different but near to each other, ideas have been reported in the management of CTS. In 1993, the American Academy of Neurology's official practice guidelines reported that in the management of CTS, noninvasive options should be the first choice and surgery should be kept for refractory cases [6]. The American Academy of Orthopedic Surgeons recommended both of the noninvasive and surgical treatments for early CTS without denervation of the median nerve. However, they also reported that an initial course should be nonoperative treatment [7].

Conservative options for CTS include nonsteroidal anti-inflammatory drugs, local steroid injection, wrist splinting, activity modification, exercises, physical therapy modalities, and alternative therapies [8]. In our clinic, routine practice is that symptomatic patients whose EMG findings reveal mild and moderate CTS findings are firstly treated with conservative approaches and we take support from physical therapy and rehabilitation department. However, patients whose EMG findings reveal severe CTS findings are advised to be operated.

The surgical options for CTS may be divided into two categories. These are endoscopic and nonendoscopic procedures. Nonendoscopic procedures may be summarized as standard open technique (conventional), a wrist-incision technique, and midpalmar-incision technique [5].

Endoscopic procedures for CTS were firstly used in 1989 and aimed at the advantage of decreased postoperative pain and faster return of patients to their work [9–11]. However, in a randomized controlled study which is comparing endoscopic surgery with open surgery for CTS, Atroshi et al. reported that endoscopic surgery was associated with less postoperative pain. However, the small size of the benefit and similarity in other outcomes make its cost effectiveness uncertain [12]. Endoscopic approach is not risk free and incomplete relief or recurrence of symptoms due to incomplete decompression. Also, injury to the superficial palmar arch or median or ulnar nerve was been reported in the literature [5, 11, 13–21]. Additionally, endoscopic procedures require costly equipment, technical support, and prolonged operative setup time [9].

The classical conventional surgery for CTS provides the surgeon full inspection of the transverse carpal ligament. Also, contents of the carpal tunnel and the presence of any “intraligamentous” motor nerve branch to the thenar muscles may be seen easily with this procedure. The optimal incision length in this procedure depends on patient- and surgeon-related factors. According to traditional definition, this surgery is performed with a longitudinal incision extending from Kaplan's cardinal line distally to beyond the wrist crease proximally, about 4-5 cm in length. However, nowadays this approach is performed with an incision 2–4 cm in length, which ends approximately 2 cm distal to the wrist crease [22]. Conventional surgery has been the mainstay in the surgical management of CTS for several decades and its success rates in terms of alleviation of patient symptoms range between 81 and 98% with minimal complications [22–27].

Wrist-incision technique is performed with a incision which is 1 cm above distal wrist crease and transverse carpal ligament is cut blindly from proximal to distal end. However, in midpalmar incision, ligament is cut distally to proximally [5, 9, 28–30]. The aim in both of these procedures is to decrease pillar pain or scar tenderness. It was reported that outcomes were similar to those of the endoscopic approach [5, 9]. In a study comparing limited palmar incision with conventional technique, authors concluded that limited palmar incision is as effective and safe as conventional technique. Moreover, they concluded that limited palmar technique had better postoperative recovery and cosmetic results [31].

In a prospective randomized study comparing limited open techniques with endoscopic procedure, authors found that results of both groups were similar to each other at the end of one year. However, patients complained of less tenderness of the scar at the second and fourth postoperative weeks in limited open release using the Strickland instrumentation group. Additionally, less thenar and hypothenar pain was reported in the same group [32].

In our study, four patients in conventional group complained of scar tenderness; however, there was no statistically significant difference. These patients benefit from local application of mupirocin containing cream. Conventional method is being performed with an incision 2–4 cm in length nowadays and this contributes to better cosmetic results [22]. In our patients, we also used an incision which was 2-3 cm in length according to patient-related factors. In our study, the most important difference between two groups was in the time of using their hands in daily activities. This point did not correlate with a study performed by Alves [33]. This difference may be due to the retrospective nature of our study.

## 4. Conclusions

1. Conventional technique always will be an effective and safe method for patients who will be operated for the first time or recurrence.
2. In patients who especially care about cosmetic results and return to work, transverse mini-incision technique may be a safe alternative in experienced hands.
